# Construction Notes

**Published:** 1906-01-06

**Authors:** 


### CONSTRUCTION NOTES.

Mrs. Umfreville Pickering has given £1,050 to the West London Hospital.

Mr. Frederick Kitchen, of Drws-y-Coed, Trefriw, Carnarvon, has left £4,650 to the Stanley Hospital, Liverpool.

Mr. Lewis James Seargeant, who died on November 28, has bequeathed £1,000 to King Edward's Hospital Fund.

The Court of the Girdlers' Company have distributed upwards of £500 amongst various hospitals and charitable institutions in London.

The Royal Orthopædic Hospital has been awarded the sum of one hundred guineas by the trustees of the late Mr. Horace Downey Harral.

A new isolation hospital, at Steppingley, near Ampthill, which provides for the numerous parishes of the Ampthill Rural District, was recently opened by the Duke of Bedford.

The Hospital of St. John and St. Elizabeth, St. John's Wood, receives a sum of £1,000 under the will of Miss Annie Russell Ferguson, to found a ward for men or to endow a bed.

The new wing which has been added to Boston Hospital was formally opened on December 22. The principal feature of the extension is in the improved sanitary arrangements which it provides.

The new Surgical Out-patient and Accident Department of Edinburgh Royal Infirmary is now completed and open to patients. It is replete with every modern equipment, and the facilities for prompt medical treatment which are provided by the increased and improved accommodation are already being felt and appreciated.

At the recent Smoke Abatement Exhibition, held under the auspices of the Royal Sanitary Institute, the silver medal was awarded to Messrs. Meldrum Brothers for their refuse-destructor, this being the only award of any kind for refuse-destructors.

